# CME ACTIVITY:* Alcaligenes xylosoxidans* Bloodstream Infections in Outpatient Oncology Office

**DOI:** 10.3201/eid1407.070616

**Published:** 2008-07

Medscape, LLC is pleased to provide online continuing medical education (CME) for this journal article, allowing clinicians the opportunity to earn CME credit. Medscape, LLC is accredited by the Accreditation Council for Continuing Medical Education (ACCME) to provide CME for physicians. Medscape, LLC designates this educational activity for a maximum of 0.5 *AMA PRA Category 1 Credits*™. Physicians should only claim credit commensurate with the extent of their participation in the activity. All other clinicians completing this activity will be issued a certificate of participation. To participate in this journal CME activity: (1) review the learning objectives and author disclosures; (2) study the education content; (3) take the post-test and/or complete the evaluation at **http://www.medscape.com/cme/eid**; (4) view/print certificate.

## Learning Objectives

Upon completion of this activity, participants will be able to:

- Identify properties of *Alcaligenes xylosoxidans*.
- Describe the clinical presentation of *A. xylosoxidans* infection in the current study.
- Specify risk factors for infection with *A. xylosoxidans*.
- Identify the primary source of infection with *A. xylosoxidans* in the current study.

## Editor

**D. Peter Drotman, MD**, Editor-in-Chief, *Emerging Infectious Diseases*. *Disclosure: D. Peter Drotman, MD, has disclosed no relevant financial relationships.*

## CME AUTHOR

**Charles P. Vega, MD**, Associate Professor; Residency Director, Department of Family Medicine, University of California, Irvine. *Disclosure: Charles P. Vega, MD, has disclosed that he has served as an advisor or consultant to Novartis, Inc.*

## AUTHORS

Disclosures: **Moon Kim, MD, MPH**; **Elizabeth Bancroft, MD, SM**; **Eleanor Lehnkering, MS**; and **Rodney M. Donlan, PhD**, have disclosed no relevant financial relationships. **Laurene Mascola, MD, MPH**, has disclosed that she has served has an advisor or consultant to Merck and MedImmune. Dr. Mascola has also disclosed that she has served as a speaker for Merck.

## Earning CME Credit

To obtain credit, you should first read the journal article. After reading the article, you should be able to answer the following, related, multiple-choice questions. To complete the questions and earn continuing medical education (CME) credit, please go to **http://www.medscape.com/cme/eid**. Credit cannot be obtained for tests completed on paper, although you may use the worksheet below to keep a record of your answers. You must be a registered user on Medscape.com. If you are not registered on Medscape.com, please click on the New Users: Free Registration link on the left hand side of the website to register. Only one answer is correct for each question. Once you successfully answer all post-test questions you will be able to view and/or print your certificate. For questions regarding the content of this activity, contact the accredited provider, CME@medscape.net. For technical assistance, contact CME@webmd.net. American Medical Association’s Physician’s Recognition Award (AMA PRA) credits are accepted in the US as evidence of participation in CME activities. For further information on this award, please refer to http://www.ama-assn.org/ama/pub/category/2922.html. The AMA has determined that physicians not licensed in the US who participate in this CME activity are eligible for *AMA PRA Category 1 Credits*™. Through agreements that the AMA has made with agencies in some countries, AMA PRA credit is acceptable as evidence of participation in CME activities. If you are not licensed in the US and want to obtain an AMA PRA CME credit, please complete the questions online, print the certificate and present it to your national medical association.

### Article Title: *Alcaligenes xylosoxidans* Bloodstream Infections in Outpatient Oncology Office

## CME Questions

1. Which of the following statements about *Alcaligenes xylosoxidans *is most accurate?
  A. *A. xylosoxidans *is gram-positive
  B. *A. xylosoxidans *is hydrophobic
  C. *A. xylosoxidans *is associated with healthcare-associated infections
  D. *A. xylosoxidans *is found only in soil
2. Which of the following statements about the clinical presentation of *A xylosoxidans* infection in the current study is most accurate?
  A. No patients with *A. xylosoxidans *infection were immunocompromised
  B. Patients with *A. xylosoxidans *were homogeneous with regard to age and clinical diagnosis
  C. Patients with *A. xylosoxidans *infection had symptoms that were attributed to side effects of chemotherapy
  D. Only 10% of patients with *A. xylosoxidans *infection were successfully treated with antibiotics
3. Which of the following factors was most different in comparing case-patients and control groups in the current study?
  A. Case-patients with *A. xylosoxidans *infection had a lower mean white blood cell count compared with controls
  B. Case-patients with *A. xylosoxidans *infection were more likely to have a central venous catheter compared with controls
  C. Case-patients with *A. xylosoxidans *infection had a greater number of intravenous medications administered compared with controls
  D. Case-patients with *A. xylosoxidans *infection had more underlying diseases compared with controls
4. Which of the following sources was most likely the primary reservoir of *A. xylosoxidans* in the current study?
  A. Tap water
  B. Mixing hood
  C. Hospital personnel
  D. Multidose vials of heparin and saline flushes

### Activity Evaluation

**Table:** 

**1. The activity supported the learning objectives.**				
Strongly Disagree				Strongly Agree
1	2	3	4	5
**2. The material was organized clearly for learning to occur.**				
Strongly Disagree				Strongly Agree
1	2	3	4	5
**3. The content learned from this activity will impact my practice.**				
Strongly Disagree				Strongly Agree
1	2	3	4	5
**4. The activity was presented objectively and free of commercial bias.**				
Strongly Disagree				Strongly Agree
1	2	3	4	5

